# Reconfiguring health purchasing for universal health coverage: insights from Nepal with relevance to low- and middle-income countries

**DOI:** 10.3389/fpubh.2025.1609219

**Published:** 2025-06-18

**Authors:** Shyamkumar Sriram, Saroj Adhikari, Bhuvan Saud

**Affiliations:** ^1^University of North Texas, Denton, TX, United States; ^2^Ministry of Health and Population, Kathmandu, Nepal; ^3^Janamaitri Foundation, Institute of Health Sciences, Lalitpur, Nepal

**Keywords:** strategic purchasing, health financing, health policy, low- and middle-income countries, institutional reform

## Abstract

Nepal’s transition to federalism and the rollout of the National Health Insurance Program (NHIP) marked critical steps toward universal health coverage. Yet, structural misalignments between health financing policies and purchasing practices persist, weakening the effectiveness of these reforms. Strategic purchasing—a key lever in health financing—has the potential to improve system efficiency, equity, and responsiveness by actively allocating pooled funds based on population needs, provider performance, and service value. However, in Nepal, legacy practices such as line-item budgeting, fragmented programmatic funding, and overlapping institutional roles hinder the adoption of this approach. The Ministry of Health and Population continues to play simultaneous roles as policymaker, purchaser, and provider, weakening accountability and the purchaser’s autonomy. Public providers are funded through both historical budgets and reimbursement schemes, diluting incentives for performance. Moreover, NHIP’s purchasing decisions are not guided by health technology assessments or cost-effectiveness analysis, raising concerns about the allocative efficiency of the benefit package. This Perspective argues that Nepal’s health financing reforms require institutional realignment, clearer role demarcation, and stronger contractual mechanisms to support strategic purchasing. Drawing on both national experience and international frameworks, it highlights the systemic disconnects and offers a way forward for embedding strategic purchasing into Nepal’s health financing architecture. By correcting these foundational misalignments, Nepal can better leverage public resources to deliver high-quality, equitable care, advancing the goals of federalism and universal health coverage simultaneously.

## Introduction

Nepal’s journey toward Universal Health Coverage (UHC) has been defined by bold policy aspirations but challenged by persistent systemic bottlenecks such as fragmented financing and overlapping roles. Following the promulgation of its federal constitution in 2015 and the establishment of the National Health Insurance Program (NHIP) in 2016, the country entered a new phase of health system reform. These developments reflected a dual commitment: to decentralize decision-making and to improve financial protection and equity in access to healthcare. They aligned with Nepal’s global obligations under the Sustainable Development Goals (SDGs) and were intended to modernize governance, increase resource mobilization, and enhance service responsiveness at all levels of care.

However, nearly a decade later, Nepal’s health sector continues to face structural inefficiencies that limit progress toward UHC. Public health spending remains low, averaging under 5% of the national budget as of 2023 (1). As a low-middle-income country, Nepal faces significant fiscal constraints owing to competing national priorities—such as infrastructure development, poverty alleviation, hunger elimination, and growing social protection responsibilities (2). These competing claims on the national budget leave little room for increased allocations to the health sector, despite rising expectations for coverage expansion and service quality improvement.

Within this constrained environment, optimizing how existing resources are spent becomes critical. This is where the concept of strategic purchasing becomes particularly relevant. Strategic purchasing refers to the deliberate allocation of pooled funds to healthcare providers based on information about population needs, provider performance, and service value. Rather than passively distributing funds through historical budgets or input-based grants, strategic purchasing uses contracts, incentives, and performance data to steer the health system toward greater efficiency, quality, and equity (3). For countries like Nepal—where out-of-pocket expenditure stands above 50% of all health spending (4) and supply-side readiness is uneven—adopting strategic purchasing is not only a technical solution but a fiscal necessity and ethical obligation.

Yet despite establishing NHIP as a national purchasing platform, Nepal has not fully embraced strategic purchasing principles. Institutional fragmentation, legacy financing practices, and blurred roles between purchasers, providers, and regulators (5) continue to dominate the system’s functioning. These problems are not unique to health. However, experience from other sectors within Nepal—such as electricity, telecommunications, and aviation—demonstrates that performance can improve dramatically when purchasing, service provision, and regulation are functionally separated. These sectors have introduced autonomous oversight bodies, formalized contractual service arrangements, and moved toward performance-based financing models. These insights offer valuable lessons for the health sector, particularly under a federal governance structure where clarity of roles and coordination is essential (6, 7).

This perspective seeks to explore why Nepal has not yet operationalized strategic purchasing, even after establishing a national insurance scheme. It argues that the current policy–practice misalignments, institutional overlaps, and weak accountability mechanisms are undermining the effectiveness of health financing reforms. Drawing on cross-sectoral lessons and international best practices, it proposes a structural rethinking of health purchasing as a strategic function. In doing so, it aims to inform ongoing debates on federalism, resource optimization, and health system reform, particularly in contexts with constrained fiscal space and ambitious UHC goals. While grounded in Nepal’s specific experience, the insights and institutional strategies discussed in this paper may hold relevance for other low-and lower-middle-income countries facing similar challenges—especially those contending with high out-of-pocket expenditure, fragmented purchasing arrangements, and limited public investment in health. Strategic purchasing, if properly institutionalized, can serve as a powerful lever for translating modest health budgets into measurable improvements in equity, efficiency, and health outcomes.

## Policy–practice misalignments undermining strategic purchasing

Nepal’s health financing landscape reflects a complex and often contradictory mix of policies, institutional structures, and legacy practices. While strategic purchasing is conceptually embedded in the establishment of the NHIP, actual implementation reveals a wide gap between aspiration and practice. This gap can be understood by examining the contradictions that arise at various levels of the health system—financing, purchasing, regulation, and service provision—across the federal, provincial, and local tiers of government.

One of the core misalignments lies in the fragmented nature of health financing. Although the NHIP was intended to be the primary vehicle for purchasing services, it accounts for only a modest share of overall health expenditure. A significant portion of funding for basic health services continues to be disbursed through conditional grants from the federal Ministry of Health and Population (MOHP), bypassing the NHIP entirely. These grants fund preventive and promotive services at local levels, including immunization and maternal health programs, which are foundational to population health (8). This parallel financing architecture undermines the NHIP’s purchasing authority, dilutes its influence over providers, and weakens its potential to function as a strategic purchaser. Without financial consolidation, providers remain accountable to multiple funding streams, often with conflicting performance expectations and reporting requirements.

Institutional fragmentation is further reinforced by overlapping mandates and blurred accountability structures. At the federal level, the MOHP continues to perform roles that span policy-making, regulation, monitoring, and—in some cases—direct service provision. Provincial and local governments, empowered under the federal constitution to manage their own health services, have also emerged as both providers and regulators, often without clear operational guidelines or delineation of responsibilities (5). This convergence of roles weakens the purchaser–provider split that is essential for strategic purchasing to function effectively. When the same institution regulates, funds, and delivers services, incentives to improve efficiency or quality become attenuated, and conflicts of interest arise that hinder objective performance assessment (9).

The design of the NHIP also contributes to misalignment. Though the National Health Insurance Board (NHIB) is legally autonomous (5), its operations are heavily constrained by centralized pricing mechanisms, uniform provider payment rates, and limited authority to enforce quality-based contracting. The same price structure is applied to both private and public hospitals, irrespective of performance or efficiency, limiting the program’s ability to use payment mechanisms as levers for improvement. Moreover, the NHIP does not currently cover preventive services or community-level interventions, excluding a large segment of health needs from its purchasing scope. This exclusion not only diminishes its relevance but also perpetuates the traditional dichotomy between preventive and curative care financing—an issue that strategic purchasing frameworks aim to overcome.

Although contracting under the NHIP incorporates the Minimum Service Standard (MSS) Program to assess service readiness, the process remains largely routine and inclusive rather than strategically selective. Any public or private hospital scoring 60 percent or above in MSS assessments becomes eligible for empanelment, yet this threshold alone is insufficient to ensure value-based service delivery or high performance. In practice, provider inclusion often hinges on availability rather than demonstrated quality, cost-effectiveness, or responsiveness to health needs (5). While the introduction of clinical and social audits—particularly in public facilities—marks a step toward accountability, their application remains inconsistent and lacks systematic feedback loops into purchasing decisions. Furthermore, the absence of an independent accreditation body weakens the institutional basis for quality assurance and continuous improvement. Crucially, Nepal also lacks a functioning health technology assessment (HTA) mechanism that could guide the inclusion of medicines, diagnostics, and interventions into the benefit package. Without HTA to appraise clinical and economic value, or an independent accreditation system to verify standards, the purchaser’s leverage to reward innovation, exclude ineffective services, or penalize underperformance remains structurally limited (10).

The fragmentation of service provision is further complicated by the existence of parallel systems of modern and traditional medicine operating under divergent policy and regulatory arrangements (5). Ayurvedic and other traditional medical institutions continue to receive public funding and function largely outside the strategic framework of the NHIP, often bypassing the same contracting, monitoring, and auditing standards applied to modern medical providers. This duality not only creates inefficiencies in resource allocation but also undermines the coherence of service quality standards and purchasing logic. The lack of integration between these systems reflects deeper structural inertia, where traditional health departments function in silos, protected by legacy policies and political patronage, rather than being rationalized or strategically aligned with national health goals.

At the sub-national level, local governments face both institutional and technical capacity constraints in planning, budgeting, and evaluating service delivery (5). Although they are legally responsible for managing health posts and primary care centers, mostly rely on federal or provincial authorities for technical support, human resources, and logistics. This dependency undermines local ownership and limits the space for innovation in service contracting or performance-based budgeting (11). Moreover, variations in capacity across local governments contribute to inequities in service access and quality, exacerbating the very disparities that strategic purchasing aims to address.

The regulatory environment further complicates matters. There is no independent regulatory authority for health service quality, price control, or accreditation. Regulatory functions are dispersed across multiple directorates and departments, many of which also serve as providers. As a result, the monitoring and evaluation of providers, especially under NHIP contracts, often lacks credibility and enforcement. In the absence of an effective regulator, the NHIB is burdened with quasi-regulatory roles for which it is neither structurally equipped nor politically empowered.

These misalignments are not merely operational glitches but systemic weaknesses that stem from the absence of a clear strategic vision for purchasing reform. The co-existence of multiple financing channels, the lack of a unified purchaser, and the failure to clearly separate regulatory, financing, and provision roles reflect a health system in transition but not yet transformed. If unaddressed, these structural issues will continue to limit the potential of the NHIP and compromise Nepal’s journey toward UHC.

## Discussion

The persistence of passive purchasing practices within Nepal’s health system reveals a deeper institutional inertia and fragmentation of governance that undermines the transformative intent of the National Health Insurance Program. Although the program represents a critical step towards Universal Health Coverage, its current design and implementation are far from aligned with the principles of strategic purchasing. The absence of performance-based contracting, coupled with uniform pricing across heterogeneous providers and the weak use of feedback mechanisms, limits the ability of the purchaser to leverage health spending for better quality, efficiency, and equity.

This misalignment is not simply a technical oversight but is embedded within the broader governance architecture of Nepal’s health sector. The simultaneous roles of the Ministry of Health and Population as policy-maker, regulator, and provider entrench a conflict of interest that discourages independent oversight. Further complicating the landscape is the overlapping authority between federal, provincial, and local governments, which weakens accountability and fosters administrative fragmentation. In this environment, even potentially valuable tools such as clinical and social audits or the Minimum Service Standard (MSS) are either underutilized or unevenly applied. Without an overarching regulatory framework—anchored in Health Technology Assessment (HTA), independent accreditation, and robust monitoring—purchasing decisions risk being reduced to procedural inclusion rather than value-based prioritization.

Experiences from other countries demonstrate that progress toward strategic purchasing requires more than technical fixes; it demands structural reforms that redefine roles and accountability pathways across stakeholders. In Thailand, for instance, the National Health Security Office operates with functional independence, resulting in 99% coverage by 2020, drawing on HTA and contracting selectively based on provider performance and cost-effectiveness (12). The United Kingdom’s National Health Service also distinguishes its commissioning functions from provider units, allowing resource allocation to be guided by explicit performance metrics and health priorities (13). These systems have evolved in political contexts different from Nepal’s, but they offer important design lessons—particularly the value of a single, autonomous purchaser with the mandate and capacity to make strategic decisions (14).

There are also instructive parallels from outside the health sector. In energy, telecommunications, and aviation, Nepal has experimented with unbundling service delivery from regulatory functions, often establishing independent regulatory authorities to ensure competition, quality, and consumer protection. These models suggest that separating the roles of purchaser, provider, and regulator is institutionally feasible even within Nepal’s federal framework, provided that legal mandates are clarified, and the necessary technical capacity is built. Such lessons underscore that institutional design matters, and that the configuration of roles and relationships among actors determines how effectively resources translate into services.

As Nepal aspires to enhance its health system’s resilience and equity, the opportunity to shift from passive to strategic purchasing remains both urgent and possible. This shift does not require abandoning existing structures but rather reconfiguring them to better support decision-making based on value, need, and performance (15). The integration of an independent HTA body, the establishment of an accreditation mechanism, and the streamlining of contracting and monitoring processes through a single purchasing window could serve as pragmatic starting points. Equally important is the political commitment to clarify the mandates of federal, provincial, and local bodies in ways that prevent role confusion and allow for effective oversight.

The existence of parallel traditional and modern medicine systems further illustrates the risks of disjointed purchasing and governance. Unless both streams are strategically aligned and brought under the same regulatory and purchasing frameworks, investments in health will continue to be diluted. Public funds must be allocated based on clear evidence of benefit, cost-effectiveness, and population need—not legacy arrangements or political considerations.

Ultimately, the transformation towards strategic purchasing in Nepal is not only a matter of health financing reform but also of governance innovation. It involves a renegotiation of relationships between the state, providers, and the public, requiring transparency, accountability, and institutional trust. Such transformation cannot be achieved through piecemeal interventions but through deliberate structural shifts supported by data, policy coherence, and sustained leadership. In moving toward this vision, Nepal must recognize that purchasing is not simply a financial transaction but a powerful policy instrument—capable of shaping provider behavior, incentivizing quality, and ensuring that the right services reach the right people at the right time.

## Conclusion

Nepal’s transition toward strategic purchasing is not about spending more, but about spending better. A clearer separation of the purchaser, provider, and regulator functions—backed by performance-linked financing and robust regulatory mechanisms—can enable smarter use of limited resources. However, implementation will require overcoming entrenched institutional overlaps, weak enforcement capacity, and a fragmented service landscape, including the coexistence of modern and traditional systems. Fiscal constraints stemming from Nepal’s lower-middle-income status and competing development priorities such as poverty alleviation, infrastructure, and food security further complicate the reform landscape. Nonetheless, gradual but decisive reforms—such as establishing an independent accreditation body, institutionalizing health technology assessment, and empowering the NHIP to act as a single strategic purchaser—can lay the foundation for a more equitable, efficient, and accountable health system. These steps would position Nepal to achieve UHC by 2030. For other low-income and lower-middle-income countries facing similar challenges—high out-of-pocket payments, limited health budgets, and institutional fragmentation—Nepal’s evolving experience offers a replicable model for realigning governance, improving value for money, and advancing strategic purchasing within constrained fiscal environments.

## Data Availability

The original contributions presented in the study are included in the article/supplementary material, further inquiries can be directed to the corresponding author.
